# Clinical and genetic characteristics and prenatal diagnosis of patients presented GDD/ID with rare monogenic causes

**DOI:** 10.1186/s13023-020-01599-y

**Published:** 2020-11-11

**Authors:** Liling Lin, Ying Zhang, Hong Pan, Jingmin Wang, Yu Qi, Yinan Ma

**Affiliations:** 1 Department of Central Laboratory, Peking University First Hospital, No. 8, Xishiku Street, Xicheng District, Beijing, 100034 China; 2 Department of Pediatrics, Peking University First Hospital, No. 8, Xishiku Street, Xicheng District, Beijing, 100034 China

**Keywords:** Global developmental delay and intellectual disability (GDD/ID), Monogenic disease, Prenatal diagnosis, Next-generation sequencing

## Abstract

**Background:**

Global developmental delay/intellectual disability (GDD/ID), used to be named as mental retardation (MR), is one of the most common phenotypes in neurogenetic diseases. In this study, we described the diagnostic courses, clinical and genetic characteristics and prenatal diagnosis of a cohort with patients presented GDD/ID with monogenic causes, from the perspective of a tertiary genetic counseling and prenatal diagnostic center.

**Method:**

We retrospectively analyzed the diagnostic courses, clinical characteristics, and genetic spectrum of patients presented GDD/ID with rare monogenic causes. We also conducted a follow-up study on prenatal diagnosis in these families. Pathogenicity of variants was interpreted by molecular geneticists and clinicians according to the guidelines of the American College of Medical Genetics and Genomics (ACMG).

**Results:**

Among 81 patients with GDD/ID caused by rare monogenic variants it often took 0.5–4.5 years and 2–8 referrals to obtain genetic diagnoses. Devlopmental delay typically occurred before 3 years of age, and patients usually presented severe to profound GDD/ID. The most common co-existing conditions were epilepsy (58%), microcephaly (21%) and facial anomalies (17%). In total, 111 pathogenic variants were found in 62 different genes among the 81 pedigrees, and 56 variants were novel. The most common inheritance patterns in this outbred Chinese population were autosomal dominant (AD; 47%), following autosomal recessive (AR; 37%), and X-linked (XL; 16%). *SCN2A, SHANK3 and STXBP1* were important causal genes. Hot-spot variants were rarely found. By the follow-up, 33 affected families, including 15, 13 and 5 families inherited in AR, AD and XL modes respectively, had undergone prenatal diagnosis. And the recurrence rates are 26.7%, 15.4% and 20% for families inherited in AR, AD, and XL patterns.

**Conclusion:**

Patients presented with GDD/ID caused by rare single gene variants are characterized by early onset, relatively severe symptoms and great clinical variability and genetic heterogeneity. Timely referrals to genetic counseling and prenatal diagnostic laboratories are important for affected families planning to have additional children.

## Introduction

Global developmental delay/intellectual disability (GDD/ID), used to be named as mental retardation (MR) is one of the most common phenotypes in neurogenetic diseases. Global developmental delay (GDD), is characterized by a delay in achieving developmental milestones in at least two of the following domains: motor skills, speech and language, cognitive skills, and social and emotional skills [1]. After growing up, many patients with GDD would demonstrate intellectual disability (ID), which is characterized by an intelligence quotient below 70 and limitations on adaptability [1, 2]. The prevalence of GDD/ID in the world population is estimated to be 1–3% [3], and the average lifetime costs (direct and indirect) to support an individual with ID have reached $1 million [4, 5].

Various environmental and genetic factors can result in GDD/ID [6]. Genetic reasons, including aneuploidy, copy number variants and single gene variants, account for 30–50% of cases [7], and Down syndrome, MECP2-related Rett syndrome and fragile X syndrome are the most common forms of genetic GDD/ID [6]. The modes of inheritance of genes related to GDD/ID include autosomal recessive (AR), autosomal dominant (AD), X-linked (XL) and mitochondrial [8]. On the basis of inherited patterns, some scholars divided those genes into ARID (autosomal recessive intellectual disability), ADID (autosomal dominant intellectual disability) and XLID (X-linked intellectual disability) [9–11]. With the improvement of next-generation sequencing (NGS) [12], monogenic genetic causes are being found more frequently in previously unexplained or idiopathic cases. To date, nearly 1334 causative genes and 1159 candidate genes have been identified as related to GDD/ID [13], and the number continues to grow.

With increasing number of patients have obtained the genetic diagnoses by NGS, it poses great challenges on subsequential genetic counseling for family reproduction plan because of our limited knowledge on most rare monogenic diseases. As effective and specific treatments for most monogenic diseases are still in development, and prenatal molecular diagnosis is an important method to prevent recurrence. Previous studies mainly focused on comparing the diagnostic yields of different NGS methods [14] on unidentified GDD/ID or expanding the phenotype and genotype spectrum of a genetic disorder or specific gene. However, the general characteristics of patients presented GDD/ID with rare monogenic causes have not been well studied. Besides, few studies concerned the genetic counseling and prenatal diagnosis.

In this article, we collected a cohort of patients with GDD/ID caused by rare single gene variants. It is the first time, to the best of our knowledge, to observed these patients from the perspective of a genetic counseling and prenatal diagnostic center. The first aim of the study was to describe the diagnostic courses and clinical and genetic spectrum of these patients. The second aim was to report prenatal molecular diagnostic results of affected families, intending to raise awareness on this area.

## Material and methods

### Study design and participants

From June 2015 to June 2019, we recruited 81 consecutive subjects under 18 years old who presented GDD/ID with rare monogenic causes. The clinical diagnosis of GDD/ID was made according to the Diagnostic and Statistical Manual of Mental Disorders, 5th edition (DSM-V) [2]. GDD was defined by delays in the achievement of motor or mental milestones in the following domains: gross and fine motor skills, speech and language, adaptability and social skills. A developmental scale for children aged 0–5 years [15] was used to assess the Developmental Quotient (DQ) for children who were under 5 years old or failed to finish the intelligence test. Patients with a DQ of less than 75 in at least two of five developmental domains were diagnosed with GDD. For patients over 5 years old, we used the Wechsler Intelligence Scale for Children (WISC) to quantify IQ. Those who had IQ scores lower than 70 and adaptability difficulties were diagnosed with ID. The tests were performed by specialists in child development.

For etiological diagnosis, according to the guideline proposed by American Psychiatric Association (APA) in 2014 [1] and Chinese Medical Association(CMA) in 2018 [16], all patients underwent systematically examinations, comprising medical history, physical examination, metabolic tests and neuroimaging study (brain MRI/CT) to exclude non genetic causes and underwent necessary genetic tests, such as G-band karyotyping, FMR1 CGG repeat testing [17], and CMA testing [18], to exclude other genetic reasons. Sanger sequencing or Trio-NGS [19, 20], including targeted exome sequencing (panel) or whole exome sequencing (WES) was performed depending on clinical judgment. The details of the detection methods are reported elsewhere.

The final clinical and genetic diagnoses were determined by a group of pediatric neurologists, clinical geneticists and molecular geneticists. The Ethics Committee of Peking University First Hospital approved the study (2020-333). Informed consent was obtained from all participants.

### Data collection

Demographic data, medical history, laboratory and genetic findings were collected. The severity of GDD/ID was classified into four groups: mild, moderate, severe and profound, defined by DQ (IQ) scores of 55–75 (50–69), 40–54 (35–49), 25–39 (20–34), and below 25 (20), respectively. The age at disease onset was calculated as the interval from the date of birth to the date when the first symptom was noticed. The age at diagnosis was calculated as the interval from the date of birth to the date when genetic diagnosis was confirmed. The interval between symptom onset and diagnosis was obtained as the age at diagnosis minus the age at disease onset. The date of genetic counseling was the time when the patient was referred to outpatient genetic counseling. The duration from genetic counseling to diagnosis was obtained by subtracting the date of diagnosis from the date of genetic counseling.

The normal standardized reference ranges of height, weight and head circumference for children at different ages were obtained from two national growth surveys of children in China [21, 22]. Microcephaly, macrocephaly, short stature and facial anomalies were defined in accordance with the Human Phenotype Ontology (HPO). Positive family history was defined as having family members who presented similar traits to the probands, with or without genetic confirmation. Abnormal birth history was defined as irregular events occurring during delivery or the neonatal period, such as amniotic fluid pollution or neonatal pathological jaundice. Abnormal prenatal ultrasound findings, such as delayed brain development, biparietal diameter anomaly and intrauterine growth retardation, were also recorded. The last follow-up was in November 2019.

### Criteria for variant interpretation

Standard gene variant nomenclature informed by the Human Genome Variation Society (HGVS) [23] was adopted to unify the description of variants. According to the 2015 American College of Medical Genetics and Genomics (ACMG) guidelines [24], variants were classified as “pathogenic”, “likely pathogenic”, “uncertain significance (VUS)”, “likely benign”, or “benign”. In order to avoid biases, patients with pathogenic or likely pathogenic variants in known genes were recruited, while patients with VUS variants in known genes or variants in candidate genes were excluded.

For genotype and phenotype comparison, we referred to the Online Mendelian Inheritance in Man (OMIM) database (https://omim.org/) and GeneReviews (https://www.ncbi.nlm.nih.gov/books/NBK1116/]). Allele frequency was searched in two population databases: the Genome Aggregation Database (GnomAD, https://gnomad.broadinstitute.org/) and the 1000 Genomes Project (1000G) [25]. The functions of missense variants were predicted in silico with the software programs SIFT [26], Polyphen-2 [27], PROVEAN [28] and MutationTaster [29]. The pathogenicity of splicing variants was predicted by the Human Splicing Finder (HSF) [30]. Co-segregation of variants was confirmed in probands and healthy parents, as well as more family members if available, via Sanger sequencing. We searched the Human Genomic Mutation Database (HGMD) [31], ClinVar [32], Ensembl (VEP) [33] and PubMed to determine whether the variant had been reported previously.

### Prenatal diagnostic testing

DNA from chorionic villi or amniotic fluid was extracted using the DNeasy Blood & Tissue Kit (Qiagen, Hilden, Germany). PCR sequencing was performed using an ABI3730 xl (Applied Biosystems, USA) to detect the causative variants harbored by probands in the family. Linkage analyses with two to five short tandem repeat (STR) markers were performed to exclude contamination with maternal DNA and confirm the originality of a variant.

### Statistical analysis

In this study, all continuous variables were found to be nonnormally distributed; accordingly, they were described as the median (lower quartile, upper quartile) values. Categorical variables were expressed as frequency rates (percentages). The chi-squared test was used to compare categorical data from at least two groups, and Fisher’s exact test was used when the samples were limited. All data analyses were performed using SPSS 23.0 software (SPSS Inc., Chicago, IL, USA). A two-sided α of less than 0.05 was used to define statistical significance.

## Results

As a tertiary genetic counseling and prenatal diagnosis center, our center served 290 families with individuals suspected of rare monogenic diseases during the 4-year study, and 142 (nearly 50%) of those patients had GDD/ID. After excluding 13 patients for missing information, 18 patients for uncertain diagnosis and 3 patients who had pathogenic variants along with atypical manifestations that could not be explained by the variants, we included a total of 108 subjects at preliminary screening. Further reviewing the case history, we excluded 16 patients who reached developmental lime stones normally in early stage and suffered GDD secondary to developmental regression, 5 patients that experienced abundant epileptiform activity in the neonatal period and then showed developmental arrest or regression and 7 patients for early death. Finally, we considered a total of 81 subjects and their core family members (Additional file 1: Fig. S1).

### Demographic features and diagnostic courses

The 81 subjects came from17 out of the 31 provinces and municipalities in mainland China. The numbers of cases inherited in AR, AD, and XL patterns were 30 (37.0%), 38 (47.0%) and 13 (16.0%), respectively. The median age was 50 months (IQR, 25–76.5), and 51 (63.0%) participants were male. The median age of onset was 3.5 months (IQR, 3–7), ranging from the day of birth to 2 years and 3 months. And 70 (95%) participants had symptoms before 1 year, all participants presented GDD before 3 years of age.

The median interval from disease onset to genetic diagnosis was 21 months (IQR, 9–55.5 m), ranging from 1 month to 12 years, and the median duration from genetic diagnosis to genetic counseling was 10 months (IQR 4–23 m; range 0–6.3y). The median number of hospital referrals was 4 (IQR 2–5; range 2–8) (Table 1 and Additional file 2: S1).

**Table 1:** Demographic features

Characteristics	N = 81
Male: Female (male%)	51: 30(63.0%)
Age, median (IQR), range, m	50 (25, 74), (2, 15y)
Age of onset, median (IQR), range, m	3.5 (3, 7), (0, 2y3m)
≤ 1 m, n (%)	9 (12.0)
1 m < age ≤ 1y, n (%)	61 (79.0)
1y < age ≤ 3y, n (%)	7 (9.0)
Age of genetic diagnosis, median (IQR), range, m	24 (14.5, 72), (3, 12y)
Interval from onset to genetic diagnosis, median (IQR), range, m	21 (9, 55.5), (1, 12y)
≤ 6 m, n (%)	13 (20.3)
6 m < t ≤ 1y, n (%)	11 (17.0)
1y < t ≤ 3y, n (%)	18 (28.0)
3y < t ≤ 5y, n (%)	10 (16.0)
> 5y, n (%)	12 (19.0)
Duration from diagnosis to genetic counseling, median (IQR), range, m	10 (4, 23), (0, 6y4m)
Number of referrals, median (IQR), range	4 (3, 5), (2, 8)
Method of diagnosis, n (%)	
Whole exome sequencing	43 (56)
Targeted exome sequencing	31 (40)
Sanger sequencing	3 (4)

IQR, interquartile range; m, months; y, years

### Clinical characteristics

Among the 81 subjects, 32 patients were diagnosed as neurodevelopmental disorders (30 syndromic ID and 2 non-syndromic ID), 20 patients had metabolic disorders, 17 patients were genetic epilepsy, and 7 patients had other neurogenetic disorders (3 neuromuscular disorders, 1 developmental brain disorder, 1 genodermatosis, and 1 multiple congenital anormaly). In addition, 54 patients had static courses (GDD had slowly improvement), 13 patients presented progressive courses (GDD followed by psychomotor regression/arrest), and 14 patients of unknown courses. The results were listed in Tables 2, 3 and Additional file 3: S2 in detail.

**Table 2:** Genetic diagnoses and pathogenic variants in 81 patients presented GDD/ID with rare monogenic causes

Patient Number	Gender	Gene (transcript)	Inheritance pattern	CDS change	Amino acid change	Zygosity	Origin	Status	Pathogenicity	Diagnosis
1	M	AKT3(NM_005465)	AD	c.1393C > T	p.Arg465Trp	het	dn	E	P	Megalencephaly-polymicrogyria-polydactyly-hydrocephalus syndrome
2	M	ALG1(NM_019109)	AR	c.77G > A	p.Trp26Ter	het	pat	E	LP	Congenital disorder of glycosylation, type Ik
				c.1197G > C	p.Glu399Asp	het	mat	N	LP	
3	F	ALG1(NM_019109)	AR	c.582 T > G	p.Cys194Trp	het	pat	N	LP	Congenital disorder of glycosylation, type Ik
				c.1079C > T	p.Ala360Val	het	mat	E	LP	
6	M	ARV1(NM_022786)	AR	c.309_310delCT	p.Cys104Hisfs*7	het	pat	N	P	Epileptic encephalopathy
				c.630_631insTA	p.Ile211Ter	het	mat	N	P	
8	F	ATP1A3(NM_152296)	AD	c.460A > G	p.Met154Val	het	dn	E	LP	Alternating hemiplegia of childhood
9	M	ATP7A(NM_000052)	XL	c.601C > T	p.Arg201Ter	het	dn	E	LP	Menkes disease
10	M	ATRX(NM_000489)	XLR	c.4626_4631delTGAAGA	p.Asp1542_Glu1543del	hemi	mat	E	LP	Mental retardation-hypotonic facies syndrome
11	M	BCS1L (NM_004328)	AR	c.245C > A	p.Ser82Ter	het	mat	E	P	Bjornstad syndrome
				c.548G > A	p.Ala183His	het	pat	E	P	
15	F	CHAMP1(NM_032436)	AD	c.1489C > T	p.Arg497Ter	het	dn	E	P	Mental retardation
16	F	CHD2(NM_001271)	AD	c.4636C > T	p.Arg1546Ter	het	dn	E	P	Epileptic encephalopathy, childhood-onset
17	F	CHD2(NM_001271)	AD	c.4173dup	p.Gln1392Thrfs*17	het	dn	E	P	Epileptic encephalopathy, childhood-onset
19	F	CPLANE1(NM_023073)	AR	c.2831G > A	p.Arg944His	het	pat	E	P	Joubert syndrome
				c.7978C > T	p.Arg2660Ter	het	mat	E	P	
21	M	DDC(NM_000790)	AR	c.1234C > T	p.Arg412Trp	het	pat	E	P	Aromatic L-amino acid decarboxylase deficiency
				c.714 + 4A > T		het	mat	E	P	
22	F	DDX3X(NM_001356)	XLR	c.1703C > T	p.Pro568Leu	het	dn	E	P	Mental retardation
23	F	DEAF1(NM_021008)	AD	c.797 T > C	p.Leu266Pro	het	dn	N	LP	Mental retardation
24	M	DIAPH1(NM_005219)	AR	c.2108dupC	p.Pro704Thrfs*71	het	pat	E	LP	Seizures, cortical blindness, microcephaly syndrome
				c.2794C > T	p.Arg932Ter	het	mat	E	LP	
25	F	DNM1(NM_004408)	AD	c.135C > A	p.Ser45Arg	het	dn	N	LP	Epileptic encephalopathy, early infantile
26	F	DOCK6(NM_020812)	AR	c.3218_3219delCT	p.Ser1073Cysfs*51	het	mat	E	P	Adams-Oliver syndrome 2
				c.4491 + 1G > A		het	pat	E	P	
27	M	ERCC8(NM_000082)	AR	c.400-2delA		het	pat	N	LP	Cockayne syndrome
				c.371_372delTG	p.Leu124GlnfsTer15	het	mat	N	LP	
28	F	FARS2(NM_006567)	AR	c.497dupC	p.Leu168Thrfs*9	het	pat	N	LP	mitochondrial encephalopathy
				c.797G > A	p.Cys266Tyr	het	mat	E	LP	
29	M	FOXG1(NM_005249)	AD	c.490A > T	p.Lys164Ter	het	dn	N	P	Rett syndrome
30	F	FOXG1(NM_005249)	AD	c.974dupT	p.Leu325Phefs*130	het	dn	N	P	Rett syndrome
31	M	GALC(NM_000153)	AR	c.466 T > C	p.Trp156Arg	het	pat	N	LP	Krabbe disease
				c.908C > T	p.Ser303Phe	het	mat	E	LP	
32	M	GAMT(NM_000156)	AR	c.212 T > G	p.Met71Arg	het	mat	N	LP	Cerebral creatine deficiency syndrome
				c.418_419delTC	p.Ser140Glyfs*50	het	pat	N	LP	
33	M	GATAD2B(NM_020699)	AD	c.941delC	p.Ser314Leufs*5	het	Pat^a^	N	P	Mental retardation
34	F	GATAD2B(NM_020699)	AD	c.1495C > T	p.Gln499Ter	het	dn	N	P	Mental retardation
35	M	GCDH(NM_000159)	AR	c.784G > A	p.Gly262Ser	het	pat	N	P	Glutaricaciduria, type I
				c.1156C > T	p.Arg386Ter	het	mat	E	LP	
36	F	GFAP(NM_002055)	AD	c.235C > T	p.Arg79Cys	het	dn	E	P	Alexander disease
37	M	GFAP(NM_002055)	AD	c.235C > T	p.Arg79Cys	het	dn	E	P	Alexander disease
38	M	GFM1(NM_024996)	AR	c.679G > A	p.Gly227Arg	het	mat	N	LP	Combined oxidative phosphorylation deficiency 1
				c.1765-2_1765-1delAG		het	pat	E	LP	
39	M	GJC2(NM_020435)	AR	c.386delC	p.Pro129Argfs*81	het	mat	N	P	Leukodystrophy, hypomyelinating;
				c.392dupC	p.His132Alafs*6	het	pat	N	P	
42	F	GLB1(NM_000404)	AR	c.1343A > T	p.Asp448Val	homo	pat/mat	E	LP	GM1-gangliosidosis, type I
44	F	GLB1(NM_000404)	AR	c.655A > C	p.Thr219Pro	het	mat	N	LP	GM1-gangliosidosis, type I
				c.1343A > T	p.Asp448Val	het	pat	N	LP	
47	M	GNB5(NM_016194)	AR	c.368C > T	p.Ser123Leu	het	pat	E	LP	Intellectual developmental disorder with cardiac arrhythmia
				c.446G > A	p.Trp149Ter	het	mat	N	LP	
48	M	GRIN2B(NM_000834)	AD	c.1963A > T	p.Ile655Phe	het	dn	N	LP	Epileptic encephalopathy, early infantile
49	M	GRIN2B(NM_000834)	AD	c.2459G > C	p.Gly820Ala	het	dn	E	LP	Mental retardation
51	M	HEXA(NM_000520)	AR	c.1510C > T	p.Arg504Cys	homo	pat/mat	E	P	Tay-Sachs disease
52	M	HEXB(NM_000521)	AR	c.1431_1432delGA	p.Lys478Thrfs*19	homo	pat/mat	N	P	Sandhoff disease
55	M	IDS(NM_000202)	XLR	c.137A > C	p.Asp46Ala	hemi	mat	E	P	Mucopolysaccharidosis II
56	M	IDS(NM_000202)	XLR	c.418 + 1G > A		hemi	mat	E	P	Mucopolysaccharidosis II
58	M	IQSEC2(NM_001111125)	XLD	c.3011 T > C	p.Leu1004Pro	hemi	dn	N	LP	Mental retardation
59	M	ISPD(NM_001101426.3)	AR	c.789 + 2 T > A		het	pat	E	LP	Walker-Warburg syndrome
				c.1251G > A	p.Gln417 = (splicing)	het	mat	N	LP	
60	M	KCNB1(NM_004975)	AD	c.1045G > T	p.Val349Phe	het	dn	N	LP	Epileptic encephalopathy
61	M	KCNQ2(NM_172107)	AD	c.1703 T > C	p.Ile568Thr	het	dn	N	LP	Epileptic encephalopathy, early infantile
62	F	KCNQ2(NM_172107)	AD	c.580A > C	p.Thr194Pro	het	dn	N	LP	Epileptic encephalopathy, early infantile
63	M	KCNT1(NM_001272003)	AD	c.667G > T	p.Val223Phe	het	dn	E	LP	Epilepsy
64	M	LRPPRC(NM_133259.3)	AR	c.2582 T > A	p.Val861Asp	het	pat	N	LP	Leigh syndrome
				c.1582 + 4A > G		het	mat	N	LP	
65	M	NAA15(NM_057175)	AD	c.63 T > G	p.Tyr21Ter	het	dn	N	P	Mental retardation
66	F	PAFAH1B1(NM_000430)	AD	c.93delT	p.Phe31Leufs*7	het	dn	N	P	Lissencephaly
67	F	PAFAH1B1(NM_000430)	AD	c.265C > T	p.Arg89Ter	het	dn	E	P	Lissencephaly
69	F	PCDH19(NM_001184880)	XL	c.1764_1765insC	p.Val589Argfs*9	het	dn	N	P	Epileptic encephalopathy
70	F	PCDH19(NM_001184880)	XL	c.445C > T	p.Pro149Ser	het	pat	N	LP	Epileptic encephalopathy
77	M	PMM2 (NM_000303)	AR	c.395 T > C	p.Ile132Thr	het	mat	E	LP	Congenital disorder of glycosylation, type Ia
				c.430 T > C	p.Phe144Leu	het	pat	E	LP	
79	M	POLR1C(NM_203290)	AR	c.325C > T	p.Arg109Cys	het	pat	E	LP	Leukodystrophy, hypomyelinating
				c.901C > T	p.Arg301Trp	het	mat	E	LP	
80	M	POMGNT1(NM_001243766)	AR	c.187C > T	p.Arg63Ter	het	pat	E	LP	Muscular dystrophy-dystroglycanopathy
				c.296 T > C	p.Leu99Pro	het	mat	N	LP	
81	M	POMT2(NM_013382)	AR	c.287A > G	p.Tyr96Cys	het	pat	E	LP	Muscular dystrophy-dystroglycanopathy
				c.551C > T	p.Thr184Met	het	mat	E	LP	
82	M	PURA(NM_005859)	AD	c.230 T > A	p.Val77Glu	het	dn	N	LP	Mental retardation
83	M	QDPR(NM_000320)	AR	c.515C > T	p.Pro172Leu	het	pat	E	LP	Hyperphenylalaninemia, BH4-deficient, C
				c.175dupT	p.Ser59Phefs*4	het	mat	N	LP	
84	M	SCN2A(NM_001040143)	AD	c.4223 T > C	p.Val1408Ala	het	dn	N	LP	Epileptic encephalopathy
85	F	SCN2A(NM_001040143)	AD	c.5144G > T	p.Gly1715Val	het	dn	N	LP	Epileptic encephalopathy
86	M	SCN2A(NM_001040143)	AD	c.5198delC	p.Pro1733Leufs*36	het	dn	N	P	Epileptic encephalopathy
88	F	SHANK3(NM_001372044.2)	AD	c.3677dup	p.Glu1227Argfs*144	het	dn	N	P	Phelan-McDermid syndrome
89	M	SHANK3(NM_001372044.2)	AD	c.3345delC	p.Gly1116Alafs*37	het	dn	N	P	Phelan-McDermid syndrome
90	M	SHANK3(NM_001372044.2)	AD	c.3904dup	p.Ala1302Glyfs*69	het	dn	E	P	Phelan-McDermid syndrome
91	M	SLC16A2(NM_006517)	XL	c.916C > T	p.Gln306Ter	hemi	mat	E	P	Allan-Herndon-Dudley syndrome
92	M	SLC9A6(NM_001177651)	XLD	c.1153C > T	p.Gln385Ter	het	dn^b^	N	P	X-linked Mental retardation
93	F	SLC9A6(NM_006359)	XLD	c.1450delC	p.Leu484Ter	het	dn	N	P	X-linked Mental retardation
94	F	SMC1A(NM_006306)	XLD	c.847_849delGAG	p.Glu283del	het	dn	N	LP	Cornelia de Lange syndrome
95	M	SOX10(NM_006941)	AD	c.395_397delCTG	p.Ala132del	het	dn	N	LP	Waardenburg syndrome
96	M	SPATA5(NM_145207)	AR	c.989_991delCAA	p.Thr330del	het	pat	E	LP	Epilepsy, hearing loss, and mental retardation syndrome
				c.1639delG	p.Ala547Leufs*18	het	mat	N	LP	
97	M	STXBP1(NM_003165)	AD	c.416C > T	p.Pro139Leu	het	dn	E	P	Epileptic encephalopathy
98	M	STXBP1(NM_003165)	AD	c.296A > C	p.Tyr99Ser	het	dn	N	LP	Epileptic encephalopathy
99	M	STXBP1(NM_003165)	AD	c.1381_1390dup	p.Arg464Glnfs*31	het	dn	N	P	Epileptic encephalopathy
101	F	SYNGAP1(NM_006772)	AD	c.1366C > T	p.Gln456Ter	het	dn	N	P	Mental retardation
102	F	SYNGAP1(NM_006772)	AD	c.1551_1552delGT	p.Tyr518Ter	het	dn	N	P	Mental retardation
105	M	TCF4(NM_001083962)	AD	c.1355_c.1356dupGG	p.Thr453Glyfs*10	het	dn	N	LP	Pitt-Hopkins syndrome
107	F	TTI2(NM_001102401)	AR	c.1100C > T	p.Pro367Leu	het	pat	E	LP	Mental retardation
				c.1402 T > C	p.Cys468Arg	het	mat	N	LP	
108	M	UBE3A(NM_130838)	AD	c.1347_1348delGA	p.Asn450Glnfs*23	het	dn	E	P	Angelman syndrome
109	F	WDR45(NM_007075)	XLD	c.519 + 1_519 + 3delGTG		hemi	dn	N	LP	Neurodegeneration with brain iron accumulation
110	M	XPA(NM_000380)	AR	c.378 T > A	p.Cys126Ter	het	mat	N	P	Xeroderma pigmentosum
				c.682C > T	p.Arg228Ter	het	pat	E	P	
111	F	ZEB2(NM_014795)	AD	c.1843C > T	p.Gln615Ter	het	dn	E	P	Mowat-Wilson syndrome

het, heterozygosity; hemi, hemizygosity; homo, homozygosity; dn, de novo; pat, paternal; mat, maternal; E, existing variant; N, novel variant; P, pathogenic; LP, likely pathogenic

^a^In patient 33, the variant was detected to be mosaic in his father’s peripheral blood

^b^In patient 92, the variant was also detected in his mother’s sample but was an extremely low peak

**Table 3:** Clinical characteristics

Characteristics	N (%)	Charicteristics	N (%)
Genetic disorders		Abnormal Brain MRI	48/70 (68.5)
Neurodevelopmental Disordrers	32/81 (39.5)	Visual impairment	10/78 (12.8)
Metabolism disorders	20/81 (24.7)	Hearing loss	4/78 (5.0)
Genetic epilepsy	17/81 (21.0)	Facial anomalies	14/81 (17.2)
Leukodystrophy	5/81 (6.2)	Head circumference anomaly	24/81 (30)
Other neurogenetic diseases	7/81 (8.4)	Microcephaly	17/81 (20.9)
Disease courses		Macrocephaly	7/81 (8.6)
Static	54/81 (66.6)	Weight	13/50 (26)
Progressive	13/81 (16.0)	Overweight	1/50 (2)
Unknown	14/81 (17.2)	Low weight	12/50 (24)
Severity of GDD/ID		Short stature	5/48 (10.4)
Mild-moderate	15/67 (22.4)	Organ involvement	13/81 (16)
Severe-profound	52/67 (77.6)	Heart	5/80 (6.0)
Unknown	14	Liver	3/80 (4.0)
Epilepsy	47/81 (58.0)	Kidney	1/80 (1.0)
Epilepsy type		Hair/skin	3/79 (3.8)
Focal	7/47 (14.9)	Bone	2/79 (2.5)
Generalized	16/47 (34.0)	Endocrine	1/81 (1.2)
Combined	14/47 (29.8)	Positive family history	5/80 (6.0)
Unknown	10/47 (21.3)	Abnormal antenatal ultrasound	10/80 (12.5)
Autism spectrum disorder	8/80 (10.0)	Abnormal birth history	4/80 (5)

GDD, global developmental delay; ID, intellectual disability

Since developmental scale should be evaluated after 3 months old, GDD might not be their first manifestations. Nearly 25% (22/81) of patients had abnormalities on appearance such as microcephalus, macrocephalus, facial anomalies, short statue, abnormal skin, hair and iris, and kyphoscoliosis, before GDD. Besides 6 patients with development and epileptic encephalopathy presented epilepsy before GDD.

Of the 81 subjects, 15 (22.4%) patients had mild-moderate GDD/ID, and the other 52 (77.6%) had severe to profound GDD/ID. The percentages of patients diagnosed by WES were 47% (7/15) in mild-moderate subjects and 60% (30/50) in severe-profound subjects, the difference of diagnostic methods used in mild and severe patients did not reach statistical significance (Table 4).

**Table 4:** Comparison of diagnostic methods between mild and severity patients

	Mild-moderate (N = 15)	Severe-profound (N = 50)	*P* value
WES	7 (46.7%)	30 (60.0%)	0.122
Panel	6 (40.0%)	19 (38%)	
Sanger	2 (13.3%)	1 (2.0%)	

WES, whole exome sequencing; panel, targeted exome sequencing

Epilepsies were co-existing in 58% (47/81) of patients. Among them, 7 patients had focal epilepsies, 16 had generalized epilepsies, 14 had combined generalized and focal epilepsies and 10 patients were classified as unknown type. Autism spectrum disorders (ASD) were confirmed in 5 patients and 3 patients had borderline ASD.

Other common presentations including facial anomalies (14 [17.2%]), microcephaly (17 [20.9%]), macrocephaly (7 [8.5%]), vision impairment (10 [12.8%]) and hearing loss (4 [5.0%]). Twelve of 60 (24%) patients had low weight, and 5/48 (10%) had short stature. Organ involvements were also observed in 16% (13/81) of subjects: 5 (6%) patients had heart involvements, 3 (4%) had liver involvements, 1 (1%) had kidney involvement, 3 (3.9%) had abnormal skin or hair manifestations, 2 had bone anomalies and 1 patient with thyroid hormone abnormality (Tables 3 and 5).

**Table 5:** Presentations of organ involvements in 13 patients

Abnormalities	N	Gene
Heart		
Patent foramen ovale	1	*ALG1*
Sick sinus syndrome	1	*GNB5*
Atrial septal defect	1	*ISPD*
Cardiomyopathy	1	*LRPPRC*
Congenital heart disease	1	*ZEB2*
Liver		
Abnormal liver function	2	*BCS1L, GLB1*
Hepatomagly	1	*GCDH*
Kidney		
Fanconi syndrome	1	*BCS1L*
Skin/Hair		
Hypopigmentation; sparse hair, twisted and partial breaks	1	*ATP7A*
Curly hair, brittle hair	1	*BCS1L*
Hypopigmented skin patch, cafe-au-lait spots, white hair	1	*SOX10*
Bone		
Spine (kyphoscoliosis); tooth(hypomature dental enamel)	1	*IDS*
Abnormal shape of skull	1	*IDS*
Endocrine		
Thyroid hormone abnormality (FT3↑, FT4↓, T4↓)	1	*SLC16A2*

In addition, 48 patients had abnormal brain imaging, the primary abnormalities including cerebral white matter changes (28 [53%]), hypoplasia of corpus callosum (12 [25%]) and cerebellar abnormalities (7 [14.6%]) (Table 6).

**Table 6:** Abnormalities of brain MRI in 48 patients

	N (%)	Gene
Cerebral cortex changes	3 (6.3)	
Lissencephaly	2 (5.2)	*PAFAH1B1(2)*
Polymicrogyria	1 (2.1)	*AKT3*
Cerebral white matter changes	28 (58.3)	
Delayed myelination	15 (31.3)	*ALG1, BCS1L, FOXG1, GLB1, GNB5, HEXA, IQSEC2, KCNB1, KCNQ2, PMM2, PURA, SHANK3, SLC16A2, TCF4, WDR45*
Hypomyelination	10 (20.1)	*ATRX, CPLANE1, DDC, GFM1, GJC2, FOLR1C, SMC1A, SOX10, STXBP1, WDR45*
Demyelination	3 (6.3)	*GALC, GFAP(2)*
Cerebral atrophy	4 (8.3)	*DOCK6, ERCC8, ISPD, SYNGAP1*
Megalencephaly	1 (2.1)	*AKT3*
Subarachnoid space enlargement	4 (8.3)	*GFM1, IDS, IQSEC2, KCNQ2, SCN2A*
Hypoplasia of corpus callosum	12 (25)	*ATRX, DDX3X, DOCK6, FOXG1, GATAD2B, IQSEC2, PAFAH1B1(2), PMM2, POLR1C, TCF4, ZEB2*
Abnormality of cerebellar	7 (14.6)	
Cerebellar atrophy	3 (6.3)	*ATP1A3, PMM2, POLR1C*
Cerebellar dysplasia	4 (8.3)	*CPLANE1, ISPD, PMM2, POLR1C*
Ventriculomegaly/hydrocephalus	6 (1.3)	*AKT3, HEXB, IDS, ERCC8, GALC, GCDH*
Basal ganglia lesions	2 (5.2)	*ALG1, FARS2*

The majority of affected individuals (94%, 75/80) were simplex cases (a single occurrence in a family), and only 5 (6%) patients had a positive family history.

Notably, 12.5% (10/80) of patients had abnormal prenatal ultrasound findings. (The details are listed in Table 7). In addition, 4 patients experienced hypoxic events during labor, the impact of hypoxic events on their development had been excluded by pediatric neurologists..

**Table 7:** Abnormalities of antenatal ultrasound in 10 patients

Abnormalities	N	Gene
Fetal growth retardation	2	*DDX3X, PMM2*
Small head circumfirence	1	*CHAMP1*
Increasing head circumfirence	1	*GFAP*
Hypoplasia of the cerebellar vermis	2	*CPLANE1, DOCK6*
Enlarged lateral ventricles	1	*SPATA5*
Hypoplasia of cerebellar vermis and dilation of lateral ventricles	1	*ISPD*
Congenital heart abnormality	1	*ZEB2*
Oligohydramnios	1	*FARS2*

### Genetic characteristics

In total, 111 pathogenic/likely pathogenic variants were found in 62 different genes among the 81 pedigrees. Of these genes, 28 genes were transmitted in the AR pattern, 25 in the AD pattern and 9 in the XL pattern. In order to analyze the disparity in genetic spectrum between different inherited models, repeated variants were included in the calculation. The results are presented in Table 8 and Additional file 4: S3 in detail. Among these disease-causing variants, there were 51 (45.9%) missense variants, 23 (20.7%) nonsense variants, 24 (21.6%) frameshift variants, 4 (3.6%) small deletion variants, 9 (8.1%) variants that caused splicing defects.

**Table 8:** Analysis of genetic spectrum

	Total
Number	111
Origin	
Paternal	32 (28.8)
Maternal	35 (31.5)
De novo	44 (39.6)
DNA change	
Substitution	77 (69.4)
Deletion	22 (19.8)
Duplication	10 (9.0)
Insertion	2 (1.8)
Amino acid change	
Missense	51 (45.9)
Nonsense	23 (20.7)
Deletion	4 (3.6)
Frameshift	24 (21.6)
Splicing defect	9 (8.1)
Loss of function	53 (47.7)
Status	
Novel	56 (50.4)
Existing	54 (48.6)

Loss of function variants include nonsense, frameshift, start lost, single or multiple exons deletion and canonical ± 1 or 2 splice sites

AR, autosomal recessive; AD, autosomal dominant; XL, X-linked

Gene ontology accumulation analyses indicated that those genes took part in multiple biological processes, including nervous system development, nervous impulse transmission, ion transport and metabolism. Genes associated with ion channel transport and nervous system development were mainly inherited in the AD model, while genes related to metabolism were mainly transmitted in AR or XL patterns (Additional file 5: Fig. S2).

Among the 62 different causative genes, *SCN2A, SHANK3* and *STXBP1* were found in 3 patients each; and *ALG1, CHD2, FOXG1, GATAD2B, GFAP, GLB1, GRIN2B, IDS, KCNQ2, PAFAH1B1, PCDH19, SLC9A6* and *SYNGAP1* in 2 patients each. The other 46 out of 62 genes were observed to have pathogenic variants only once each in this cohort.

Most variants were unique in this cohort, while two variants were relatively common. One was the c.1343 A > T in the *GLB1* gene, which occurred in 3 alleles of 2 patients (patient 42/44) among 2 patients with *GLB1*-related diseases.Iit was a high frequency variant in *GLB1* [34]. The other was a de novo variant c.235C > T in *GFAP*, which was detected in two unrelated patients (Nos.36 and 37) with Alexander Disease. It was a variant that had been reported several times [35–37] but absent in the Normal Population Database (GnomAD and 1000G). Additionally, a homozygous substitution variant, c.1510C > T, was found in patients (Nos. 51) with Tay-Sachs disease, confirmed by hexosaminidase A enzyme deficiency (< 1.1 nmol/mg/h). Multiple studies [38–40] have reported the pathogenicity of these variants, suggesting that the 1510th base pair in the coding sequence of *HEXA* (NM_000520) was a common variant position.

Notably, 56 (50.4%) variants were identified as novel variants, and 54 (48.6%) variants have been included in disease databases (ClinVar or HGMD) or reported in PubMed articles. The rate was similar to that in previous studies [41–45]. The proportions of novel variants in ARID, ADID and XLID were 43.8%, 65% and 50%, respectively. This suggests that variant spectrum in known ID genes have not been fully explored in all inheritance patterns. The higher rate of novel variants in ADID might be explained by the fact that most variants arose de novo in the AD pattern.

The major difference among ARID, ADID and XLID lies in the origin of variants. Of the 30 patients with ARID, 27 (90%) patients carried compound heterozygous variants, and 3 (10%) patients harbored homozygous variants. We confirmed that in all patients, the two abnormal alleles were separately inherited from healthy outbred parents who carried the heterozygous variants. Among 38 patients with ADID, 37 (97.4%) variants arose de novo, 1 variant was transmitted from a mosaic father. Of the 13 patients with XLID, 7 (53.8%) patients (2 males, 5 female) had de novo variants, 5 male patients harbored hemizygous variants inherited from their asymptomatic heterozygous mother, and 1 female (patient 70) inherited the heterozygous variant c.445C > T in *PCDH19* from her non-symptomatic father. This unique characteristic was supported by previous reports [46].

In addition, parental somatic mosaicism was found in 2 cases. One is patient 33, who presented with facial dysmorphism and GDD, had a c.941del in *GATAD2B*. The variant was also detected at a low frequency in his paternal peripheral blood genomic DNA but absent in samples of his healthy mother and sister. Therefore, it is likely that the father carries somatic and germline mosaicism for this variant. The other is patient 93, who harbored a hemizygous c.1153C > T in SLC9A6. And his mother was suspected to have the variant in mosaic state with a low peak in her peripheral blood Sanger sequencing.

### Prenatal diagnostic results

By the time of follow-up, totally 33 families underwent prenatal tests to determine whether the next child would harbor the same pathogenic variants as the index patient in the fetal period. As demonstrated in Table 9 and Additional file 6: S4, among them, 15 cases were ARID, 13 cases were ADID and 4 were XLID. And 28 (84.8%) patients chose amniocentesis, and 5 (15.2%) patients underwent chorionic villus sampling. Among the 15 AR cases, 4 fetuses were found to carry two pathogenic variants that originated from parents who were healthy carriers, 9 fetuses harbored one variant, and 2 fetuses did not have any variants. Among the 13 AD cases, 11 fetuses did not have the variants, while 2 fetuses carried the same variants as the proband in the *GATAD2B* gene. Of the 5 XL cases, only 1 fetus harbored the pathogenic variant. The recurrence rates of AR, AD and XL modes were 26.7%, 15.4% and 20% respectively. All variants carried by fetuses were verified after birth or induction of labor.

**Table 9:** Results of prenatal diagnosis

	Total	AR	AD	XL
Number of patients	33	15	13	5
Pregnancy status at counseling				
Not pregnant	19 (57.5)	11 (73.3)	7 (53.8)	1 (20.0)
Pregnant	14 (42.5)	4 (26.7)	6 (46.2)	4 (80.0)
Sample				
Amniotic fluid	28 (84.8)	12 (80.0)	12 (92.3)	4 (80.0)
Chorionic villus	5 (15.2)	3 (20.0)	1 (7.7)	1 (20.0)
Number of variants carried by the fetus				
2	4 (12.1)	4 (26.7)	–	–
1	12 (36.4)	9 (60.0)	2 (15.4)	1 (20.0)
0	17 (51.5)	2 (13.3)	11 (84.6)	4 (80.0)

AR, autosomal recessive; AD, autosomal dominant; XL, X-linked

The appropriate time for genetic counseling is before the next pregnancy, owing to the additional procedure to confirm original molecular tests. In this study, 14 (42.5%) families had been pregnant before referral to genetic counseling and prenatal diagnosis, which might influence further management. It was suggested that, for most families, referral to genetic counseling is usually delayed and reflected a shortage of related resources. Therefore, timely genetic counseling after index patients obtain a genetic diagnosis, should be emphasized to families who have reproduction plan.

## Discussion

In this article, we analyzed the diagnostic courses, clinical and genetic characteristics, and prenatal diagnosis of 81 individuals with GDD/ID of monogenic origin. It often took 0.5–4.5 years and 2–8 referrals to obtain a genetic diagnosis after disease onset, reflecting the difficulty of diagnosis. Many factors are associated with this difficulty, including genetic heterogeneity, phenotype and penetrance variability, and shared signs and symptoms. Despite the great variability, when treated as a group of diseases, some features are noteworthy. The empirical findings regarding onset age, severity and coexisting symptoms can be summarized as follows.

One of the distinguishing features is the early age of onset. In our study, all individuals presented developmental delay before 3 years of age, and 80% of them showed abnormal symptoms in the first year of life. Nearly 10% of patients had abnormal during the prenatal stage. This finding is in accordance with previous studies showing that in monogenic forms of ID, the time of onset ranges from the 12th week after conception to early childhood [8, 47]. It also implies that future efforts should be made using NGS in the prenatal stage to detect abnormal prenatal ultrasound findings available and affordable [48, 49].

The severity of GDD/ID ranged from mild to profound in our study, and about 80% of patients had severe to profound disability. This finding is consistent with previous reports that GDD/ID caused by genetic factors could be more severe than those resulting from environmental factors, as the latter are usually mild [50, 51]. Previous studies concluded that de novo variants in ADID genes are the major causes of severe ID, and ARID and XLID are rare in outbred European or Korean populations [41–44], and ARID with homozygous variants is most prevalent in consanguineous populations [45]. In this outbred Chinese cohort, we found that ARID, ADID and XLID had similar rates of severe cases (81%, 80%, and 60%). In addition, ARID with compound heterozygous variants and ADID accounted for approximately 32% and 35.8% of severe cases. Therefore, our results suggested that ARID with compound heterozygous variants also plays an important role in patients with GDD/ID caused by monogenic origin in outbred population. The inconsistency could be partially explained by the difference in study design and population. Another possible explanation is that each person carries 100 to 200 heterozygous private variants that are potentially deleterious [52], and when asymptomatic and unrelated parents carry such a variant in the same ARID gene, their offspring have a 25% chance of illness.

Most patients (over 95%) had other symptoms besides GDD/ID. Approximately 58% of subjects encompassed epilepsy and 10% had ASD. This finding supports the theory that GDD/IDs share a common etiology with other cognitive and neurological disorders including ASD and seizures [50]. In addition, 43.2% (35/81) of patients manifested abnormalities in appearance, such as microcephalus (21%), macrocephalus (8.6%), facial anomalies (17.2%), short stature (10.4%), and changes in skin or hair (3.8%). The percentage of patients in our cohort who had involvement in other organs, including the heart, kidney, liver, bone and endocrine, was under 16%, which might be lower than the rate in GDD/ID caused by aneuploid.

Genetic heterogeneity was prominent among these patients. Patients with different variants in the same gene could have different manifestations, while patients could have similar phenotype even with different causal genes. For example, the clinical presentation of three patients (No. 88, 89, 90) who were diagnosed with Phelan-McDermid syndrome and carried different frameshift variants in SHANK3 were not exactly similar. Patient 88 and 89 presented profound speech delay and ASD, while facial dysmorphism was found only in patient 88 and epilepsy was found only in patient 89. Patient 90 showed moderate developmental delay and facial dysmorphism but did not have epilepsy or ASD. This finding is consistent with previous studies intended to discover the phenotype-genotype correlation of Phelan-McDermid syndrome [53, 54].

Although experts recommended that when a genetic disorder was highly suspected, the first tier genetic testing is to detect the specific gene directly. We observed that most of our patients were diagnosed with the help of NGS rather than specific gene detection. It suggested the strong power of NGS in diagnosing patients with GDD/ID. We also observed that the percentages of patients diagnosed by WES or panel were similar in this cohort, while WGS is currently not widely used in domestic clinical situations. The best NGS technique for screening patients with GDD/ID remained controversial. It was reported that the diagnostic yields of panel, WES, and WGS in unexplained GDD/ID were up to 11–32%, 40%, and 42% respectively [43, 44, 55–57]. Previously opinions considered that targeted NGS has the priorities of deeper coverage depth and lower cost than WES, however, hardly any targeted ID-panel could cover a great number of ID genes and chase up to the speed of the discovering new candidate genes, as it is estimated that the number of ID genes is over 1000 and still uprising. Therefore, the choice of personalized diagnostic method relies on the clinicians’ experience and varies from person to person.

The second aim of our study was to analyze the prenatal diagnostic situation of these groups of patients. Among the 33 families experienced prenatal diagnosis, the recurrence rates are 26.7%, 15.4% and 20% for ARID, ADID, and XLID, respectively. For GDD/ID caused by variants in autosomal genes, the recurrence rate is determined by whether the origin of the variants is inherited from parents or occurring de novo. For XLID, the recurrence rate is related not only to the originality of the variants but also to the sex of the fetus, which should be taken into consideration in specific situations. The necessity for prenatal diagnosis of variants inherited from parents has reached consensus in ARID and recessive XLID.

However, with an estimated recurrence rate less than 1%, the question of whether it is necessary to perform prenatal diagnosis for de novo variants in ADID and XLID remains controversial. In this cohort, 2 families with ADID tested positive in prenatal diagnosis, suggesting the high possibility of parental mosaicism in these cases. However, the mosaicism was confirmed in only one (50%) AD family via Sanger sequencing with peripheral blood. Therefore, the recurrence rate of de novo variant in AD families reached 8.3% (1/12), much higher than 1%.

Multiple lines of evidence suggested that the occurrence of parental germline mosaicism is underestimated. Firstly, while previous studies suggested that both Sanger sequencing and exome sequencing have the ability to detect somatic mosaicism [44, 51]. Due to the limitation of sequencing depth and difficulty in specimen acquisition, the existence of mosaicism in asymptomatic parents, which results in an increasing recurrence rate, is usually undetectable at present. Studies using deep amplicon sequencing and digital PCR methods to detect multiple samples found that the proportion of parental mosaicism in some AD or XLID genes reached 5–20% [58–60]. Secoundly, nearly half of the ADID and XLID genes in this study have related case reports on germline mosaicism (Additional file 7: S5). Therefore, whether confirmation of parental mosaicism or not, we recommend that prenatal diagnosis is also necessary for de novo variants in ADID and XLID situations. In conclusion, genetic counseling and prenatal diagnostic services are important for families with any ARID, ADID and XLID probands.

Another important observation is that at present, genetic counseling and prenatal diagnostic services are not timely for nearly 50% of families, who were first referred for genetic counseling only after conceiving again. This might result from the lack of awareness and limitation of resources in this field. With the improvement and availability of genetic testing technology, an increasing number of individuals will obtain accurate genetic diagnoses. Additionally, with the implementation of a universal two-child policy [61], the need for genetic counseling and prenatal diagnosis is bound to increase. Therefore, more attention should be paid to this area.

The strengths of our study could be summarized as following. Firstly, it is the first article, to report the general characteristic of a group of patients presented GDD/ID with rare monogenic causes. Secondly, our observations on diagnostic time and methods could be set as baseline data and compared with the that obtained 5–10 years later. Thirdly, via calculating the recurrence rate in a group of families affected by AR, AD and XL genes, our research revealed that the recurrence rate of de novo variant might be underestimated in real world practice. Finally, it is the first retrospective cohort study observed from the perspective of genetic counseling and prenatal diagnostic center, and our result reflected the lack of awareness and shortage of resources in this field.

There are several limitations in our study. One is that rare monogenic neurogenetic diseases with GDD/ID consist of a group of different disorders, and while analyzing it in a cohort, we failed to perform genotype–phenotype correlation of a single syndrome or gene. However, to delineate the relationship in detail, a group of individuals with variants in the same gene or diagnosed with the same syndrome are needed. This kind of study is restricted by sporadic cases. Additonally, our understanding of these rare monogenic diseases is insufficient, and regular follow-up observations of such patients might provide additional clinical information. Futhermore, as it is a single center study, the results would be influenced by selection bias.

## Conclusion

In summary, individuals with GDD/ID caused by rare monogenic variants are characterized by early onset, relatively severe phenotype as well as great clinical variability and genetic heterogeneity. Patients or pedigrees with such features should be considered to undergo appropriate NGS as early as possible. The spectrum of causal genes and pathogenic variants has not yet been fully discovered. Therefore, clinicians, genetic counselors and genetic laboratories should collaborate tightly to address the problems of diagnosis posed by the bewildering clinical and genetic heterogeneity. Moreover, obtaining clinical and genetic diagnosis is not the final step; timely referral to genetic counseling and prenatal diagnostic laboratories are important for families that plan to have additional children.

## Supplementary information

**Additional file 1:** **Fig. S1.** Flow diagram for inclusion and exclusion of studies.

**Additional file 2:** **S1.** Demographic data.

**Additional file 3:** **S2.** Clinical characteristics.

**Additional file 4:** **S3.** Variant spectrum and interpretation.

**Additional file 5:** **Fig. S2.** Genes that were reported to have parental germline mosaicism cases.

**Additional file 6:** **S4.** Prenatal diagnosis.

**Additional file 7:** **S5.** REViGO tree analysis of GO terms obtained with DAVID.

## Data Availability

Most related data and material have been uploaded as supplementary. For more detailed information, please contact the corresponding author.

## References

[1] Moeschler JB, Shevell M (2014). Committee on G: comprehensive evaluation of the child with intellectual disability or global developmental delays. Pediatrics.

[2] American Psychiatric Association (2013). Diagnostic and statistical manual of mental disorders.

[3] Maulik PK, Mascarenhas MN, Mathers CD, Dua T, Saxena S (2011). Prevalence of intellectual disability: a meta-analysis of population-based studies. Res Dev Disabil.

[4] Department of information, evidence and research. WHO methods and data sources for global burden of disease estimates 2000–2016. 2018.

[5] Centers for Disease Control and Prevention (CDC). Economic costs associated with mental retardation, cerebral palsy, hearing loss, and vision impairment—United States. MMWR Morb Mortal Wkly Rep. 2004; 53(3):57–59. 14749614

[6] Flore LA, Milunsky JM (2012). Updates in the genetic evaluation of the child with global developmental delay or intellectual disability. Semin Pediatr Neurol.

[7] Srour M, Shevell M (2014). Genetics and the investigation of developmental delay/intellectual disability. Arch Dis Child.

[8] Chiurazzi P, Pirozzi F (2016). Advances in understanding—genetic basis of intellectual disability. F1000Res.

[9] Neri G, Schwartz CE, Lubs HA, Stevenson RE (2018). X-linked intellectual disability update 2017. Am J Med Genet A.

[10] Wieczorek D (2018). Autosomal dominant intellectual disability. Med Genet.

[11] Jamra R (2018). Genetics of autosomal recessive intellectual disability. Med Genet.

[12] Harripaul R, Noor A, Ayub M, Vincent JB (2017). The use of next-generation sequencing for research and diagnostics for intellectual disability. Cold Spring Harb Perspect Med.

[13] Kochinke K, Zweier C, Nijhof B, Fenckova M, Cizek P, Honti F, Keerthikumar S, Oortveld MA, Kleefstra T, Kramer JM (2016). Systematic phenomics analysis deconvolutes genes mutated in intellectual disability into biologically coherent modules. Am J Hum Genet.

[14] Srivastava S, Love-Nichols JA, Dies KA, Ledbetter DH, Martin CL, Chung WK, Firth HV, Frazier T, Hansen RL, Prock L (2019). Meta-analysis and multidisciplinary consensus statement: exome sequencing is a first-tier clinical diagnostic test for individuals with neurodevelopmental disorders. Genet Med.

[15] Mushi Z, Lingying F, Xiangyun L, Xiu X, Huirong L, keli W. Standardization of the mental developmental screening test (DST) for children aged 0~6 years in China. Zhonghua Er Ke Za Zhi. 1997;35(03):117–117.

[16] Subspecialty Group of Neurology, Chinese Society of Pediatrics, Chinese Medical Association; Project Expert Group of Childhood Neuropathy, China Neurologist Association. Experts' consensus on the diagnostic strategies of etiology for intellectual disability or global developmental delay in children. Zhonghua Er Ke Za Zhi. 2018;56(11):806–810. 10.3760/cma.j.issn.0578-1310.2018.11.003 30392203

[17] Chen X, Wang J, Xie H, Zhou W, Wu Y, Wang J, Qin J, Guo J, Gu Q, Zhang X (2015). Fragile X syndrome screening in Chinese children with unknown intellectual developmental disorder. BMC Pediatr.

[18] Yi Z, Pan H, Li L, Wu H, Wang S, Ma Y, Qi Y (2016). Chromosome Xq28 duplication encompassing MECP2: clinical and molecular analysis of 16 new patients from 10 families in China. Eur J Med Genet.

[19] Yan H, Shi Z, Wu Y, Xiao J, Gu Q, Yang Y, Li M, Gao K, Chen Y, Yang X (2019). Targeted next generation sequencing in 112 Chinese patients with intellectual disability/developmental delay: novel mutations and candidate gene. BMC Med Genet.

[20] Chen J, Che L, Xu C, Zhao S, Yang J, Li M, Li G, Shen Y (2020). Cardio-facio-cutaneous syndrome-associated pathogenic MAP2K1 variants activate autophagy. Gene.

[21] Coordinating Study Group of Nine Cities on the Physical Growth and Development of Children, Pediatrics CIo: A national survey on growth of children under 7 years of age in nine cities of China. Chin J Pediatr 2007; 45(8):609–614. 18021536

[22] Hui L, Cheng-ye J, Xin-nan Z, Ya-qin Z (2009). Height and weight standardized growth charts for Chinese children and adolescents aged 0 to 18 years. Zhonghua Er Ke Za Zhi.

[23] den Dunnen JT, Dalgleish R, Maglott DR, Hart RK, Greenblatt MS, McGowan-Jordan J, Roux AF, Smith T, Antonarakis SE, Taschner PE (2016). HGVS recommendations for the description of sequence variants: 2016 update. Hum Mutat.

[24] Richards S, Aziz N, Bale S, Bick D, Das S, Gastier-Foster J, Grody WW, Hegde M, Lyon E, Spector E (2015). Standards and guidelines for the interpretation of sequence variants: a joint consensus recommendation of the American College of Medical Genetics and Genomics and the Association for Molecular Pathology. Genet Med.

[25] Genomes Project C, Auton A, Brooks LD, Durbin RM, Garrison EP, Kang HM, Korbel JO, Marchini JL, McCarthy S, McVean GA (2015). A global reference for human genetic variation. Nature.

[26] Kumar P, Henikoff S, Ng PC (2009). Predicting the effects of coding non-synonymous variants on protein function using the SIFT algorithm. Nat Protoc.

[27] Adzhubei IA, Schmidt S, Peshkin L, Ramensky VE, Gerasimova A, Bork P, Kondrashov AS, Sunyaev SR (2010). A method and server for predicting damaging missense mutations. Nat Methods.

[28] Choi Y, Sims GE, Murphy S, Miller JR, Chan AP (2012). Predicting the functional effect of amino acid substitutions and indels. PLoS ONE.

[29] Schwarz JM, Cooper DN, Schuelke M, Seelow D (2014). MutationTaster2: mutation prediction for the deep-sequencing age. Nat Methods.

[30] Desmet F-O, Hamroun D, Lalande M, Collod-Béroud G, Claustres M, Béroud C (2009). Human Splicing Finder: an online bioinformatics tool to predict splicing signals. Nucleic Acids Res.

[31] Stenson PD, Mort M, Ball EV, Evans K, Hayden M, Heywood S, Hussain M, Phillips AD, Cooper DN (2017). The Human Gene Mutation Database: towards a comprehensive repository of inherited mutation data for medical research, genetic diagnosis and next-generation sequencing studies. Hum Genet.

[32] Landrum MJ, Lee JM, Benson M, Brown GR, Chao C, Chitipiralla S, Gu B, Hart J, Hoffman D, Jang W (2018). ClinVar: improving access to variant interpretations and supporting evidence. Nucleic Acids Res.

[33] Cunningham F, Achuthan P, Akanni W, Allen J, Amode MR, Armean IM, Bennett R, Bhai J, Billis K, Boddu S (2019). Ensembl 2019. Nucleic Acids Res.

[34] Hofer D, Paul K, Fantur K, Beck M, Roubergue A, Vellodi A, Poorthuis BJ, Michelakakis H, Plecko B, Paschke E (2010). Phenotype determining alleles in GM1 gangliosidosis patients bearing novel GLB1 mutations. Clin Genet.

[35] Caroli F, Biancheri R, Seri M, Rossi A, Pessagno A, Bugiani M, Corsolini F, Savasta S, Romano S, Antonelli C (2007). GFAP mutations and polymorphisms in 13 unrelated Italian patients affected by Alexander disease. Clin Genet.

[36] Brenner M, Johnson AB, Boespflug-Tanguy O, Rodriguez D, Goldman JE, Messing A (2001). Mutations in GFAP, encoding glial fibrillary acidic protein, are associated with Alexander disease. Nat Genet.

[37] Li R, Johnson AB, Salomons G, Goldman JE, Naidu S, Quinlan R, Cree B, Ruyle SZ, Banwell B, D'Hooghe M (2005). Glial fibrillary acidic protein mutations in infantile, juvenile, and adult forms of Alexander disease. Ann Neurol.

[38] Raghavan SS, Krusell A, Krusell J, Lyerla TA, Kolodny EH (1985). GM2-ganglioside metabolism in hexosaminidase A deficiency states: determination in situ using labeled GM2 added to fibroblast cultures. Am J Hum Genet.

[39] Akli S, Chelly J, Lacorte JM, Poenaru L, Kahn A (1991). Seven novel Tay-Sachs mutations detected by chemical mismatch cleavage of PCR-amplified cDNA fragments. Genomics.

[40] Paw BH, Wood LC, Neufeld EF (1991). A third mutation at the CpG dinucleotide of codon 504 and a silent mutation at codon 506 of the HEX A gene. Am J Hum Genet.

[41] Gieldon L, Mackenroth L, Kahlert AK, Lemke JR, Porrmann J, Schallner J, von der Hagen M, Markus S, Weidensee S, Novotna B (2018). Diagnostic value of partial exome sequencing in developmental disorders. PLoS ONE.

[42] de Ligt J, Willemsen MH, van Bon BW, Kleefstra T, Yntema HG, Kroes T, Vulto-van Silfhout AT, Koolen DA, de Vries P, Gilissen C (2012). Diagnostic exome sequencing in persons with severe intellectual disability. N Engl J Med.

[43] Han JY, Jang JH, Park J, Lee IG (2018). Targeted Next-generation sequencing of korean patients with developmental delay and/or intellectual disability. Front Pediatr.

[44] Martinez F, Caro-Llopis A, Rosello M, Oltra S, Mayo S, Monfort S, Orellana C (2017). High diagnostic yield of syndromic intellectual disability by targeted next-generation sequencing. J Med Genet.

[45] Kahrizi K, Hu H, Hosseini M, Kalscheuer VM, Fattahi Z, Beheshtian M, Suckow V, Mohseni M, Lipkowitz B, Mehvari S (2019). Effect of inbreeding on intellectual disability revisited by trio sequencing. Clin Genet.

[46] Samanta D (2020). PCDH19-Related Epilepsy Syndrome: A Comprehensive Clinical Review. Pediatr Neurol.

[47] Tau GZ, Peterson BS (2010). Normal development of brain circuits. Neuropsychopharmacology.

[48] Lord J, McMullan DJ, Eberhardt RY, Rinck G, Hamilton SJ, Quinlan-Jones E, Prigmore E, Keelagher R, Best SK, Carey GK (2019). Prenatal exome sequencing analysis in fetal structural anomalies detected by ultrasonography (PAGE): a cohort study. Lancet.

[49] Petrovski S, Aggarwal V, Giordano JL, Stosic M, Wou K, Bier L, Spiegel E, Brennan K, Stong N, Jobanputra V (2019). Whole-exome sequencing in the evaluation of fetal structural anomalies: a prospective cohort study. Lancet.

[50] van Bokhoven H (2011). Genetic and epigenetic networks in intellectual disabilities. Annu Rev Genet.

[51] Rauch A, Wieczorek D, Graf E, Wieland T, Endele S, Schwarzmayr T, Albrecht B, Bartholdi D, Beygo J, Di Donato N (2012). Range of genetic mutations associated with severe non-syndromic sporadic intellectual disability: an exome sequencing study. The Lancet.

[52] Mefford HC, Batshaw ML, Hoffman EP (2012). Genomics, intellectual disability, and autism. N Engl J Med.

[53] De Rubeis S, Siper PM, Durkin A, Weissman J, Muratet F, Halpern D, Trelles MDP, Frank Y, Lozano R, Wang AT (2018). Delineation of the genetic and clinical spectrum of Phelan-McDermid syndrome caused by SHANK3 point mutations. Mol Autism.

[54] Kolevzon A, Delaby E, Berry-Kravis E, Buxbaum JD, Betancur C (2019). Neuropsychiatric decompensation in adolescents and adults with Phelan-McDermid syndrome: a systematic review of the literature. Mol Autism.

[55] Grozeva D, Carss K, Spasic-Boskovic O, Tejada MI, Gecz J, Shaw M, Corbett M, Haan E, Thompson E, Friend K (2015). Targeted next-generation sequencing analysis of 1,000 individuals with intellectual disability. Hum Mutat.

[56] Redin C, Gerard B, Lauer J, Herenger Y, Muller J, Quartier A, Masurel-Paulet A, Willems M, Lesca G, El-Chehadeh S (2014). Efficient strategy for the molecular diagnosis of intellectual disability using targeted high-throughput sequencing. J Med Genet.

[57] Wright CF, FitzPatrick DR, Firth HV (2018). Paediatric genomics: diagnosing rare disease in children. Nat Rev Genet.

[58] Xu X, Yang X, Wu Q, Liu A, Yang X, Ye AY, Huang AY, Li J, Wang M, Yu Z (2015). Amplicon resequencing identified parental mosaicism for approximately 10% of "de novo" SCN1A mutations in children with dravet syndrome. Hum Mutat.

[59] Liu A, Yang X, Yang X, Wu Q, Zhang J, Sun D, Yang Z, Jiang Y, Wu X, Wei L (2019). Mosaicism and incomplete penetrance of PCDH19 mutations. J Med Genet.

[60] Zhang Q, Yang X, Wang J, Li J, Wu Q, Wen Y, Zhao Y, Zhang X, Yao H, Wu X (2019). Genomic mosaicism in the pathogenesis and inheritance of a Rett syndrome cohort. Genet Med.

[61] Zeng Y, Hesketh T (2016). The effects of China's universal two-child policy. The Lancet.

