# Supplementary material for: Clinical and genetic characteristics and prenatal diagnosis of patients presented GDD/ID with rare monogenic causes
Source: Orphanet J Rare Dis. 2020 Nov 11;15:317. doi: 10.1186/s13023-020-01599-y (PMC7656751; doi:10.1186/s13023-020-01599-y)
Supplement: Supplementary file 1 — Additional file 1: Fig. S1. Flow diagram for inclusion and exclusion of studies. [file 13023_2020_1599_MOESM1_ESM.docx]

### Additional file 1: Fig. S1. Flow Diagram for Inclusion and Exclusion of Studies.

GDD, global developmental delay; ID, intellectual disability; P, pathogenicity; LP, likely pathogenicity; VUS, variant of uncertain significance; DD, developmental delay; AR, autosomal recessive; AD, autosomal dominant; XL, X-link.
