# Supplementary material for: Clinical and genetic characteristics and prenatal diagnosis of patients presented GDD/ID with rare monogenic causes
Source: Orphanet J Rare Dis. 2020 Nov 11;15:317. doi: 10.1186/s13023-020-01599-y (PMC7656751; doi:10.1186/s13023-020-01599-y)

**Additional file 5: Fig. S2. REViGO tree analysis of GO terms obtained with DAVID.** Panel a shows BP terms associated with the 62 ID genes; Panel b shows BP terms associated with the 28 ARID genes; Panel c shows BP terms associated with the 25ADID genes; Panel d shows BP terms associated with the 9 XLID genes. DAVID, Database for Annotation Visualization, and Integrated Discovery; GO, Gene Ontology; BP, Biological Process; REViGO, Reduce + Visualize Gene Ontology.

a


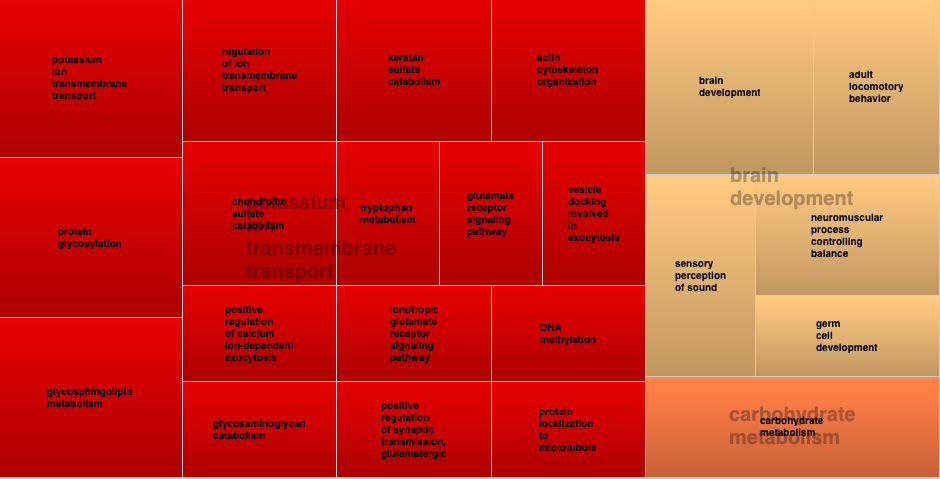


b


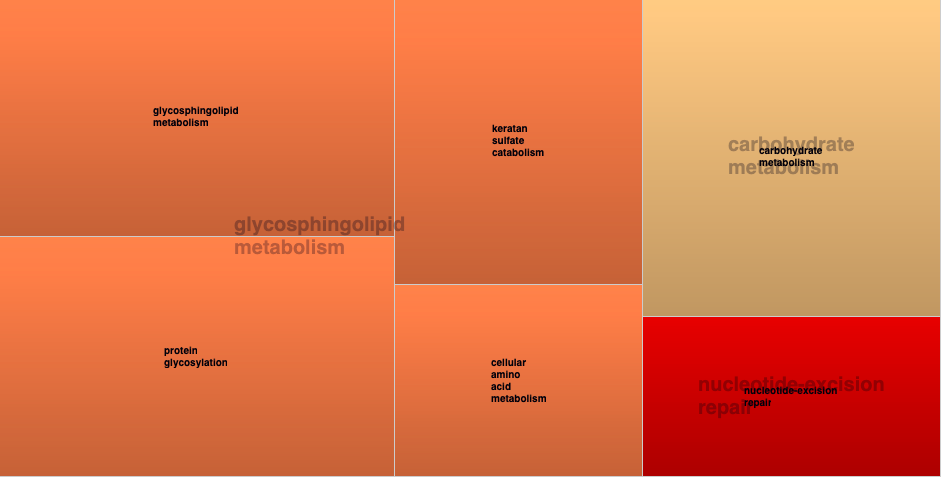


c


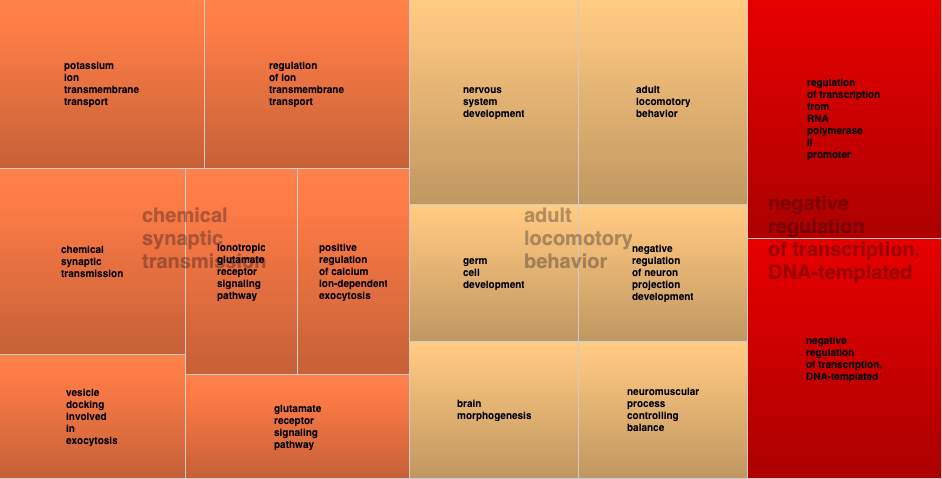


d


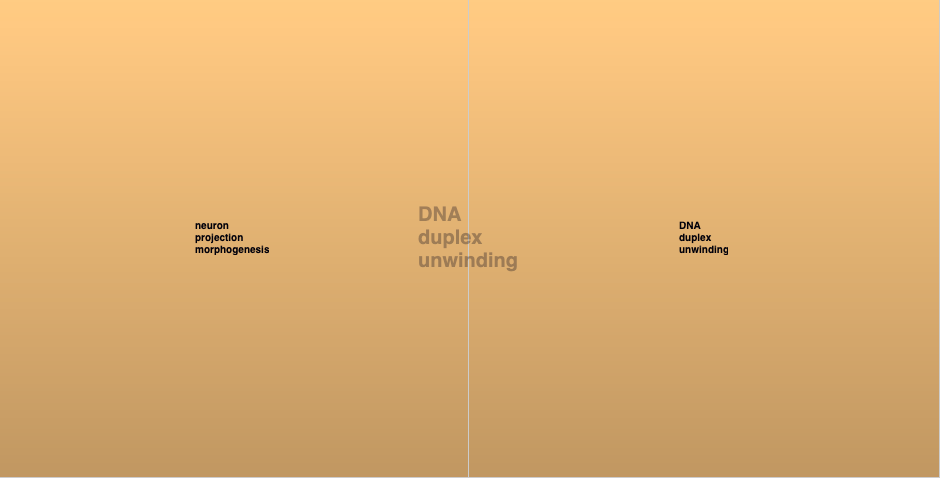

Supplement: Supplementary file 5 — Additional file 5: Fig. S2. Genes that were reported to have parental germline mosaicism cases. [file 13023_2020_1599_MOESM5_ESM.docx]
