# Supplementary material for: Clinical and genetic characteristics and prenatal diagnosis of patients presented GDD/ID with rare monogenic causes
Source: Orphanet J Rare Dis. 2020 Nov 11;15:317. doi: 10.1186/s13023-020-01599-y (PMC7656751; doi:10.1186/s13023-020-01599-y)
Supplement: Supplementary file 7 — Additional file 7: S5. REViGO tree analysis of GO terms obtained with DAVID. [file 13023_2020_1599_MOESM7_ESM.docx]

**Additional file 7: S5.** **Genes that were reported to have parental germline mosaicism cases.**

Table S5-1. Twelve out of twenty-five autosomal dominant genes that have germline mosaicism cases.

| No. | Gene symbol | PMID |
| --- | --- | --- |
| 1 | *AKT3* | 32446860 |
| 2 | *ATP1A3* | 27726050 |
| 3 | *CHD2* | 28960266 |
| 4 | *FOXG1* | 26364767 |
| 5 | *KCNQ2* | 22275249 |
| 6 | *KCNT1* | 30234941 |
| 7 | *SCN2A* | 24814476 |
| 8 | *SHANK3* | 25646853  20385823 |
| 9 | *SOX10* | 17352389 |
| 10 | *SYNGAP1* | 30789692 |
| 11 | *UBE3A* | 16100729  24311297 |
| 12 | *ZEB2* | 16088920  18230842 |

Table S5-2. Seven out of nine X-linked genes that have germline mosaicism cases.

| No. | Gene symbol | PMID |
| --- | --- | --- |
| 1 | *ATRX* | 10602370 |
| 2 | *DCX* | 11175293 |
| 3 | *DDX3X* | 31274575 |
| 4 | *IDS* | 26762690  20301451 |
| 5 | PCDH19 | 21519002 |
| 6 | *SMC1A* | 22581668  23505322  20301283 |
| 7 | *WDR45* | 26577041 |
